# Reliability and validity of the Thai version of the Pediatric Quality of Life inventory™ 3.0 Duchenne Muscular Dystrophy module in Thai children with Duchenne Muscular Dystrophy

**DOI:** 10.1186/s12955-019-1140-y

**Published:** 2019-05-02

**Authors:** Apirada Thongsing, Surachai Likasitwattanakul, Oranee Sanmaneechai

**Affiliations:** Division of Neurology, Department of Pediatrics, Faculty of Medicine Siriraj Hospital, Mahidol University, 2 Wanglang Road, Bangkoknoi, Bangkok, 10700 Thailand

**Keywords:** Health-related quality of life (HRQOL), Duchenne muscular dystrophy, DMD, PedsQL, Thai, Psychometric properties

## Abstract

**Background:**

Duchenne Muscular Dystrophy (DMD) is the most common genetic neuromuscular disorder in children. This chronic illness may impact the physical, family, social and school life of affected children and their families. These impacts can be assessed using a disease-specific measure of health-related quality of life (HRQOL). The Pediatric Quality of Life Inventory™ (PedsQL™) 3.0 DMD Module is designed to assess quality of life in children with DMD. This study aimed to evaluate the reliability and validity of the Thai version of the PedsQL™ 3.0 DMD Module in Thai children aged 5–18 years.

**Method and materials:**

The Thai translation of the PedsQL™ 3.0 Duchenne Muscular Dystrophy Module was performed in accordance with established guidelines using forward-back translation and was approved by the creator of the instrument. The Thai version of the scale was administered to children with DMD and their parents at the neuromuscular clinic at Siriraj Hospital and during the annual DMD Day meeting. Psychometric properties were established, and a re-test was performed within 2–4 weeks.

**Results:**

Fifty-six children were enrolled. An acceptable level of internal reliability was achieved, as measured by α > 0.7 (total score: child report α = 0.88, parent report α = 0.92). Test-retest reliability showed good agreement, with the following intraclass correlation coefficients (ICCs) for the total score (calculated using all subscales from the child reports and parent reports): child report ICCs = 0.74 and parent report ICCs = 0.88. The mean total scale score was 66.03 for ambulatory children and 55.87 (*P* = 0.08) for non-ambulatory children according to child self-reports and 70.01 (ambulatory) and 54.29 (non-ambulatory) (*P* ≤ 0.01) according to parent proxy reports. The child self-reports were in acceptable agreement with the parent proxy reports for most subscales (ICC range 0.49–0.81).

**Conclusions:**

The PedsQL™ 3.0 DMD Module Thai version is a reliable and valid measure of disease-specific health-related quality of life in Thai children with Duchenne muscular dystrophy.

## Introduction

Duchenne muscular dystrophy (DMD) is the most common genetic neuromuscular disorder in children. The reported incidence of DMD ranges from 10.71 to 27.78 per 100,000 [1]. It is a chronic progressive illness that results in the loss of proximal muscle motor function. Declining motor function leads to a considerable physical, psychological and financial burden for both affected children and their families. Quality of life (QoL) refers to all aspects of life, including non-health related issues, while health-related QoL (HRQoL) focuses on the impacts that illness and treatment may have on QoL [2]. HRQoL is an important outcome assessment in disease progression evaluation, clinical trials and research in pediatric populations with chronic health conditions [3]. Since disease progression in DMD has major impacts on patients and their families, HRQoL measurement is important for understanding and assessing difficulties that require professional intervention [4]. There are multiple HRQoL questionnaires with both generic and disease-specific versions [5]. Generic HRQoL measures are important for assessing and comparing outcomes across different populations and interventions, while disease-specific HRQoL measures assess the special states and concerns of specific diagnostic groups. The Pediatric Quality of Life Inventory™ (PedsQL™) 4.0 Generic Core Scales questionnaire yields information on the physical, emotional, social, and school functioning of children during the previous 4 weeks [6]. The PedsQL™ 3.0 DMD Module is designed to assess quality of life in children with DMD from 5 to 18 years old [7]. The PedsQL™ DMD 3.0 module has 4 scales assessing the ‘Daily Activities’, ‘Treatment Barriers’, ‘Worry’, and ‘Communication’ of the child during the previous 4 weeks [7]. Disease-specific measures provide enhanced measurement sensitivity for specific chronic health condition [8]. Thus, both generic and disease-specific measures should be administered to pediatric patients with chronic diseases for a holistic HRQoL assessment [9, 10]. Currently, the Thai PedsQL™ 4.0 Generic Core Scale is available for the general population, but the translation and reliability and validity assessments of the PedsQL™ 3.0 DMD Module in the Thai language have not been completed. To fully evaluate the health-related quality of life of DMD patients, we administered both the disease-specific and generic modules.

## Objective

We aimed to accurately translate the PedsQL™ 3.0 DMD Module into the Thai language, evaluate the reliability and validity of the Thai version, and determine the PedsQL™ DMD 3.0 module score in Thai children aged 5–18 years with DMD.

## Methods

### Study design and patient population

A cross-sectional study was performed on 56 children aged 5–18 years with DMD (confirmed by either genetic study or muscle biopsy) who were evaluated at the neuromuscular clinic of Siriraj Hospital and during the annual DMD Day meeting between 2016 and 2017. This study was approved by the Siriraj Institutional Review Board (SIRB) committee. Children were excluded from this study if they had other chronic diseases. Informed consent and assent were obtained. We calculated the sample size by estimating that the child-parent agreement ICC of the total score would be 0.5 ± 0.2 with 95% CI; the resulting sample size was *n* = 56 (the child-parent Agreement ICC reported in a previous study [7] ranged from 0.279–0.681).

### Measures and procedures

The PedsQL™ 3.0 DMD Module consists of 18 items in 4 domains: ‘Daily Activities’ (5 items), ‘Treatment Barriers’ (4 items), ‘Worry’ (6 items) and ‘Communication’ (3 items). The child self-report questionnaire is available for two age groups: 8–12 years (children) and 13–18 years (teens). The parent proxy report questionnaire is available for 3 age groups: 5–7 years (young children), 8–12 years and 13–18 years. The questionnaire is answered using a 5-point response scale to indicate how much of a problem each item has been in the past month (0 = never a problem to 4 = almost always a problem). Items are reverse scored and linearly transformed to a 0–100 scale (0 = 100; 1 = 75; 2 = 50; 3 = 25; 4 = 0), so that higher scores indicate better HRQoL. The translation process was included in the creator’s approval of the study. Thai translation of the PedsQL™ 3.0 DMD Module was performed according to established linguistic translation guidelines [11]. All steps were completed, and the final version was accepted by the MAPI Research Institute in Lyon, France, on behalf of Dr. James W. Varni, the creator and copyright owner of the PedsQL™. The Thai version of the scale was administered to children with DMD and their caregivers separately. For children who were unable to read, a research assistant read the questionnaire aloud and recorded the child’s responses using the response scale. Demographic and clinical manifestation data were reviewed. The retest was performed within 2–4 weeks during a routine clinical visit or by paper post mail.

### Data analysis

Data were analyzed with SPSS (Statistical Package for the Social Sciences) version 2.0 with the *P*-value set at ≤0.05. The demographic data of the patients and caregivers were reported as percentages, means, standard deviations and ranges. The feasibility of the questionnaire was assessed using the percentage of missing data [3, 12–14]. The percentage of scores at the extremes of the scaling range, that is, the maximum possible score (ceiling effect) and the minimum possible score (floor effect), were determined [15]. Surveys with small floor or ceiling effects (≤15%) are considered to have acceptable measurement standards, while surveys with moderate floor or ceiling effects (> 15%) are considered less precise measurements of latent constructs at the extremes of the scale [16]. The internal consistency reliability of the Thai version scale was determined at the first evaluation by calculating Cronbach’s alpha coefficient [17]. Scales with reliabilities ≥0.70 are considered satisfactory. The item-subscale correlations for the Thai version were determined at baseline using Pearson correlation analysis. Good scaling is achieved if the correlation between an item and its hypothesized subscale is stronger than its correlation with other subscales. The test-retest reliability of the Thai version of the scale was assessed for a subset of the sample (*n* = 33) using intraclass correlation coefficients (ICCs) [18]. Intraclass correlations range from − 1 to 1, with higher values indicating better agreement. ICCs ≤0.40 were designated as indicating poor to fair agreement, 0.41–0.60 as moderate agreement, 0.61–0.80 as good agreement, and 0.81–1.00 as excellent agreement [19–21]. Agreement between child self-reports and parent proxy reports for the Thai version of the scale was determined using ICCs [22]. Construct validity was assessed between ambulatory and non-ambulatory children and between children who were receiving steroids and those who were not receiving steroids using the independent sample t-test to compare first evaluation scores. We compared the PedsQL™ DMD Module scales by age group using independent-sample t tests for the child self-reports, since they included two age groups, and using analysis of variance methods with Tukey’s correction for multiple comparisons for the parent proxy reports, which included three age groups.

## Results

### Demographic and clinical characteristics

A total of 56 male DMD patients from 51 families agreed to participate. The median age of the patients was 11.7 years (range 5 to 18). The demographic characteristics of the DMD patients are shown in Table 1.

**Table 1:** Demographic characteristics of 56 DMD patients

Data	Mean ± SD (range) or N (%)
Age at onset (years)	5.42 ± 2.08 (1–9)
Age at time of the evaluation (years)	11.71 ± 3.59 (5–18)
5–7	9 (16.1)
8–12	23 (41.1)
13–18	24 (42.9)
Non-ambulatory patients	34 (60.7)
Age when patient became non-ambulatory (years)	10.09 ± 2.02 (7–17)
Current steroid use	40 (71.4)
Family history	13 (23.2)

### Feasibility

The percentage of missing child self-report responses at the item level was 3.57%. Two non-ambulatory children (ages 12 and 17 years) could not complete the child self-report questionnaire due to intellectual disability. The parent report was completed for these two patients. The percentage of missing data for the parent-report questionnaire was 1.79%.

### Reliability

#### Internal consistency reliability

The internal consistency reliability of the scale was determined at the first evaluation by calculating Cronbach’s alpha coefficient. All self-report subscales and proxy-report subscales exceeded the minimum reliability standard of 0.7 (Table 2). There was a ceiling effect for the parent proxy responses to the communication subscale.

**Table 2:** Internal consistency reliability of the Thai version of the PedsQL™ 3.0 DMD Module

Scale (# items)	n	Cronbach’s alpha	Mean	SD	%floor	%ceiling
Child self-report						
Total (18)	45	0.88	58.80	17.80	0	0
Daily Activities (5)	45	0.79	52.33	23.87	2.2	0
Treatment Barriers (4)	45	0.80	64.21	23.46	2.2	4.4
Worry (6)	45	0.86	63.24	24.75	2.2	6.7
Communication (3)	45	0.85	53.52	26.44	4.4	6.7
Parent proxy report						
Total (18)	55	0.92	60.58	20.55	0	0
Daily Activities (5)	55	0.87	51.91	26.22	3.6	3.6
Treatment Barriers (4)	55	0.82	66.02	23.81	1.8	12.7
Worry (6)	55	0.84	60.15	23.17	1.8	1.8
Communication (3)	55	0.95	68.64	32.23	7.3	30.9

#### Item-subscale correlations

The Pearson correlation coefficients between the items and the subscale scores are presented in Table 3. We found that most items had moderate to strong correlations with their hypothesized subscales; these were higher than the items’ correlations with the other subscales (*P* < 0.05).

**Table 3:** Item-subscale correlations of the Thai version of the PedsQL™ 3.0 DMD module for DMD

Subscales & Items	Child				Parent			
	A	T	W	C	A	T	W	C
Daily Activities								
Trouble eating with a fork and knife	0.76^a^	0.39	0.44	0.00	0.77^a^	0.47	0.34	0.27
Hard to write or draw with a pen or pencil	0.74^a^	0.51	0.40	0.21	0.80^a^	0.70	0.44	0.58
Hard to put on my clothes	0.84^a^	0.17	0.02	0.02	0.90^a^	0.52	0.34	0.25
Hard to use the toilet without help	0.74^a^	0.20	0.10	0.04	0.81^a^	0.51	0.21	0.36
Need more time than others to complete tasks	0.64^a^	0.30	0.19	0.01	0.75^a^	0.50	0.38	0.24
Treatment								
Hard to take medicines	0.24	0.60^a^	0.45	0.30	0.31	0.60^a^	0.49	0.37
Physical therapy or daily stretching hurts	0.48	0.81^a^	0.41	0.45	0.62	0.85^a^	0.38	0.48
Hard to be responsible for my medicines or physical therapy	0.27	0.88^a^	0.44	0.41	0.57	0.86^a^	0.49	0.43
Hard to manage my muscle problem	0.29	0.83^a^	0.61	0.34	0.61	0.88^a^	0.60	0.45
Worry								
Worry about my muscle problem	0.24	0.58	0.78^a^	0.02	0.59	0.74	0.76^a^	0.39
Worry whether or not my medicines are working	0.15	0.52	0.81^a^	0.10	0.23	0.38	0.76^a^	0.33
Worry about my family	0.49	0.48	0.83^a^	0.18	0.37	0.44	0.73^a^	0.28
Worry about needing help from others	0.30	0.39	0.80^a^	0.19	0.38	0.40	0.84^a^	0.40
Worry about not being accepted by others	0.19	0.32	0.73^a^	0.42	0.22	0.45	0.71^a^	0.45
Worry about being treated differently from others my age	0.05	0.47	0.68^a^	0.37	0.06	0.29	0.68^a^	0.34
Communication								
Hard for me to tell the doctors and nurses how I feel	0.00	0.37	0.21	0.87^a^	0.36	0.47	0.42	0.97^a^
Hard for me to ask the doctors and nurses questions	0.05	0.40	0.20	0.91^a^	0.46	0.58	0.52	0.96^a^
Hard for me to explain my muscle problem to other people	0.16	0.48	0.29	0.84^a^	0.40	0.47	0.44	0.93^a^

Note: Pearson’s product moment correlations are designated as small (0.10), medium (0.30), and large (0.50)

A Daily Activities, T Treatment, W Worry, C Communication

^a^ Values represent correlations between items and their hypothesized subscales

#### Test-retest reliability

A subset of children (*n* = 33) and parents (*n* = 40) completed the PedsQL™ DMD Module 3.0 measures a second time 2–4 weeks later during a routinely scheduled clinic visit or by mail (Table 4). The ICCs for test–retest reliability showed good to excellent agreement for most scales for the child self-report questionnaire and for all scales for the parent proxy-report questionnaire.

**Table 4:** Test-retest reliability of the Thai version of the PedsQL™ 3.0 DMD Module

Scale (# items)	Intraclass correlation coefficient, ICC (95% CI)
Child self-report	
Total (18)	0.74 (0.54–0.87)
Daily Activities (5)	0.88 (0.77–0.94)
Treatment Barriers (4)	0.63 (0.37–0.80)
Worry (6)	0.53 (0.23–0.74)
Communication (3)	0.54 (0.25–0.75)
Parent proxy-report	
Total (18)	0.88 (0.76–0.93)
Daily Activities (5)	0.90 (0.81–0.95)
Treatment Barriers (4)	0.82 (0.68–0.90)
Worry (6)	0.66 (0.43–0.81)
Communication (3)	0.68 (0.47–0.82)

#### Parent-child agreement

Agreement between the parent and child responses was good to excellent for two of the four subscales (‘Daily Activities’ and ‘Treatment Barriers’, ICC 0.81 each) and moderate for the subscales ‘Worry’ (ICC 0.57) and ‘Communication’ (ICC 0.49) (Table 5).

**Table 5:** Parent-child agreement of the Thai version of the PedsQL™ 3.0 DMD Module

Scale (# items)	Intraclass correlation coefficient, ICC (95% CI)
Total (18)	0.76 (0.61, 0.86)
Daily Activities (5)	0.81 (0.68, 0.89)
Treatment Barriers (4)	0.81 (0.68, 0.89)
Worry (6)	0.57 (0.33, 0.74)
Communication (3)	0.49 (0.22, 0.68)

### Construct validity

Construct validity was assessed between the ambulatory and non-ambulatory patients and between those who received steroids and those who did not receive steroids using the independent samples t-test to compare the scores from the first evaluation (Tables 6 and 7). The means for all domains were higher for ambulatory patients and for subjects receiving steroids. The ‘Daily Activities’ domain score was significantly correlated with ambulatory status for both the child self-report and parent proxy-report questionnaires. The ‘Total score’, ‘Treatment Barriers’, and ‘Communication’ subscale scores were also significantly related to mobility on the parent proxy-report questionnaires. The total score and the ‘Treatment Barriers’ and ‘Daily Activities’ subscale scores were significantly related to steroid use for both the child self-report and parent proxy-report questionnaires.

**Table 6:** Construct validity of the Thai version of the PedsQL™ 3.0 DMD Module, known-groups method comparing ambulatory and non-ambulatory patients

Scale (# items)	Ambulatory		Non-ambulatory		P	Difference (95% CI)
	Mean	SD	Mean	SD		
Child self-report	(*N* = 13)		(*N* = 32)			
Total (18)	66.03	18.54	55.87	16.91	0.08	10.16 (−1.37, 21.69)
Daily Activities (5)	67.69	17.51	46.09	23.48	< 0.01	21.60 (7.02, 36.18)
Treatment Barriers (4)	70.19	21.52	61.78	24.10	0.28	8.41 (−7.12, 23.94)
Worry (6)	66.03	25.16	62.11	24.90	0.64	3.92 (−12.65, 20.48)
Communication (3)	57.69	26.23	51.82	26.75	0.51	5.87 (−11.78, 23.52)
Parent proxy report	(*N* = 22)		(*N* = 33)			
Total (18)	70.01	15.56	54.29	21.25	< 0.01	15.72 (5.12, 26.32)
Daily Activities (5)	67.95	17.57	41.21	25.74	< 0.001	26.74 (14.13, 39.36)
Treatment Barriers (4)	75.28	18.75	59.85	25.05	0.02	15.43 (2.87, 28.00)
Worry (6)	63.07	19.17	58.21	25.59	0.45	4.86 (−7.98, 17.70)
Communication (3)	80.30	27.76	60.86	33.04	0.03	19.44 (2.30, 36.59)

**Table 7:** Construct validity of the Thai version of the PedsQL™ 3.0 DMD Module, known-groups method comparing patients receiving steroids to patients not receiving steroids

Scale (# items)	Steroid use		No steroid use		P	Difference (95% CI)
	Mean	SD	Mean	SD		
Child self-report	(*N* = 33)		(*N* = 12)			
Total (18)	62.26	16.75	49.37	17.81	0.03	12.95 (1.38, 24.53)
Daily Activities (5)	57.42	23.29	38.33	20.26	0.02	19.09 (3.76, 34.42)
Treatment Barriers (4)	69.57	21.77	49.48	22.37	0.01	20.09 (5.19, 35.00)
Worry (6)	65.78	23.98	56.25	26.56	0.26	9.53 (−7.23, 26.30)
Communication (3)	53.54	28.26	53.47	21.75	0.99	0.06 (−18.12, 18.25)
Parent proxy report	(*N* = 39)		(*N* = 16)			
Total (18)	64.74	19.82	50.43	19.22	0.02	14.31 (2.61, 26.01)
Daily Activities (5)	58.21	25.27	36.56	22.41	< 0.01	21.64 (7.05, 36.23)
Treatment Barriers (4)	70.19	22.51	55.86	24.52	0.04	14.33 (0.58, 28.09)
Worry (6)	63.03	24.28	53.13	19.09	0.15	9.91 (−3.75, 23.56)
Communication (3)	71.79	33.26	60.94	29.14	0.26	10.86 (−8.28, 30.00)

### Differences in PedsQL™ DMD scores between age groups

The mean scores on the PedsQL™ DMD by age group are displayed in Table 8. The child self-report mean score for daily activities was significantly higher for the 8- to 12-year-old group than for the 13- to 18-year-old group (*P* = 0.03). Parents also reported significantly lower Daily Activities scores for the 13- to 18-year-old group than for the 8- to 12-year-old group (P = 0.03) and the 5- to 7-year-old group (*P* = 0.004). Parent reports yielded significantly higher total scores (better overall QOL) for the 5- to 7-year-old group compared with the 13- to 18-year-old group (P = 0.03). Parents reports yielded significantly higher Communication scores (indicating better communication about the disease) for the 5- to 7-year-old group compared with the 8- to 12-year-old group (*P* = 0.047).

**Table 8:** Comparison of PedsQ™ 3.0 DMD Module scores by age group

Scale (# items)	Age 5–7 years		Age 8–12 years		Age 13–18 years	
	Mean	SD	Mean	SD	Mean	SD
Child self-report			(*N* = 22)		(*N* = 23)	
Total (18)	–	–	61.49	20.63	56.23	14.61
Daily Activities (5)	–	–	60.00^*a^	23.09	45.00	22.71
Treatment Barriers (4)	–	–	67.05	26.74	61.50	20.07
Worry (6)	–	–	64.96	27.54	61.59	22.26
Communication (3)	–	–	49.62	30.80	57.25	21.51
Parent proxy report	(*N* = 9)		(*N* = 23)		(*N* = 23)	
Total (18)	75.31^*a^	11.74	60.75	23.70	54.65	17.28
Daily Activities (5)	70.56^*a^	16.09	57.61^*a^	25.58	38.91	24.21
Treatment Barriers (4)	81.25	18.22	66.58	25.67	59.51	21.72
Worry (6)	67.59	15.42	59.42	27.13	57.97	21.61
Communication (3)	90.74^*b^	15.28	60.87	36.88	67.75	29.01

Higher scores equal better health-related quality of life (fewer symptoms or problems)

-, data not available; * *P* < 0.05

^a^ Significantly different than ages 13–18 years

^b^ Significantly different than ages 8–12 years

## Discussion

This study provides evidence that the PedsQL™ 3.0 DMD Module Thai version is a valid and reliable instrument for evaluating quality of life among the pediatric DMD population. There were few missing item responses, indicating that the children and their parents were able to provide good-quality data. The few missing item responses on the self-report questionnaires were due to intellectual disability. The missing responses on the parent proxy report questionnaires could be due to misunderstanding the instructions provided. No floor effects were found for the subscales. However, a ceiling effect was seen for the communication subscale on the parent proxy report questionnaire, suggesting that these parents had good communication with their children.

The PedsQL™ 3.0 DMD Module Thai version showed acceptable values that exceeded the minimum alpha coefficient standard of 0.70 for internal consistency on all subscales for both child self-report and parent proxy report, similar to the results for the original version [7]. Thus, the Thai PedsQL™ 3.0 DMD Module can be regarded as an internally consistent instrument. The item-subscale score correlations were analyzed using Pearson correlations. The correlations between items and their hypothesized subscales were higher than the items’ correlations with other subscales, demonstrating good scaling for the child self-reports and parent proxy reports.

Responses to the Thai version scale were in good to excellent agreement for most subscales across a 2–4 week time period and were significantly correlated, indicating test-retest reliability. Parent-child agreement showed that the concordance between the perceptions of parents and their sons was good to excellent for all but the ‘Worry’ and ‘Communication’ subscales. Our study showed that children with DMD worried less about their health problems than their parents do, which is different from the findings of a previous study [7]. This could be due to the fact that parents generally understand disease progression and severity more than children do. The parents were mainly concerned with their child’s muscular problems. In addition, we believe that response bias is unlikely to explain this finding because the subjects did not know the research assistant conducting the interview. Our study found that the children reported more communication difficulty than parents. Urzark and colleagues also reported poor to moderate agreement between children with DMD and their parents, suggesting that evaluating both perspectives should be standard practice [7]. Bray and colleagues also found moderate to poor agreement between 35 parents and their sons with DMD [23]. Child self-report scores and parent proxy report scores often show less than optimal agreement in HRQOL questionnaires of children with and without chronic illness [24, 25].

Known group comparisons (ambulatory vs non-ambulatory and steroids vs no steroids) were used to assess construct validity and demonstrated that the instrument is able to discriminate between groups. The total score and the scores for the ‘Treatment Barriers’ and ‘Daily Activities’ subscales were significantly related to steroid use in both the child self-reports and the parent proxy reports. This finding may be explained by the effect of steroids on slowing disease progression. The ‘Daily Activities’ subscale scores for the child self-report responses was significantly related to ambulatory status. The subscales ‘Treatment Barriers’, ‘Worry’ and ‘Communication’ in the child self-report group showed unqualified statistical discriminative abilities, while the ‘Daily Activities’, ‘Treatment Barriers’ and ‘Communication’ subscales in the parent proxy report group were significantly related to ambulatory status, similar to a 2012 study from the USA [7]. Interestingly, the communication subscale score in the parent proxy report group was significantly higher for the ambulatory group, although in theory this domain should be the least affected by ambulatory status. It is possible that the older age of the children in the non-ambulatory group (14 vs 8 years) enabled them to understand the disease more and that this, along with psychological changes in the teenage years, resulted in withdrawal from communication, as perceived by parents. However, this finding may also suggest an underlying problem of depression due to limited activity that requires further psychosocial evaluation. The parent proxy and child scores were significantly different only for the daily activities domain. This could be due to the small sample size. Further testing with larger sample sizes and more extensive evaluation of clinical data, such as LVEF function and the need for respiratory support, are merited.

The mean total score of 58.80 and the mean of all subscales in the child self-report group were lower than those reported in a previous study from the USA [7], although the mean age of the children (11.7 years in the Thai study vs 10.4 years in the USA study) and the proportion of non-ambulatory patients (60.7% in the Thai study vs 58% in the USA study) were similar. The lower mean scores observed in our study could be due to poorer access to medical resources, lower incomes, and the limited access to public transportation and other services for handicapped people in Thailand.

With advancing age, the boys reported a significant decrease in daily activities, which correlated with the parent proxy reports (Table 8). As the disease progress, children will experience more weakness, which results in restrictions in daily activities. This result is similar to the results of a previous study from the USA [7]. Interestingly, the parent proxy-report showed better communication in the 5- to 7-year-old group compared to the 8- to 12-year-old group. This could result from the fact that younger children have not yet experienced complex medical needs, so it may be easier for them to explain their health-related problems.

There are several limitations of our study. Some of our children were limited by intellectual disability, which reduced our sample size. A larger sample would have enhanced the factor analysis. Factor analysis would enable us to further explore the construct validity and dimensionality of the instrument [8]. We did not administer the PedsQL™ Generic Core Module to our DMD population; thus, we could not determine the inter-correlation between the DMD module and the generic core scale. We did not evaluate responsiveness, which is used to detect HRQOL changes over time and can be regarded as additional evidence of instrument validity [3]. We also could not perform IQ tests for all patients due to a lack of resources.

## Conclusion

The PedsQL™ 3.0 DMD Module Thai version has acceptable reliability and validity. It is important to assess HRQoL from the perspective of both children and their parents. Our patient population had lower scores than a similar cohort from the USA, suggesting that it is important to consider environmental modifications to minimize the impact of the physical disability seen in DMD. The PedsQL™ 3.0 DMD Module Thai version can be used as an outcome measure in clinical practice and research. Further study with a larger DMD population is needed to conduct factor analysis.

## Abbreviations

DMD: Duchenne muscular dystrophy
HRQoL: Health-related quality of life
ICC: Intraclass correlation coefficient
PedsQL™: Pediatric Quality of Life Inventory™
SIRB: Siriraj Institutional Review Board
SPSS: Statistical Package for the Social Sciences
USA: United States of America
